# Real-world outcomes of PD-L1 inhibitors combined with thoracic radiotherapy in the first-line treatment of extensive stage small cell lung cancer

**DOI:** 10.1186/s13014-023-02308-2

**Published:** 2023-07-04

**Authors:** Jianfeng Peng, Lemeng Zhang, Liping Wang, Hui Feng, Dongmei Yao, Rui Meng, Xiaomei Liu, Xiaohua Li, Ningbo Liu, Bingxu Tan, Zhaoqin Huang, Shanshan Li, Xiangjiao Meng

**Affiliations:** 1 Department of Radiation Oncology, Shandong Cancer Hospital and Institute, Shandong First Medical University and Shandong Academy of Medical Sciences, Jiyan Road 440, Jinan, 250117 Shandong China; 2 Department of Thoracic Department, Hunan Cancer Hospital, Changsha, China; 3 Department of Medical Oncology, Baotou Cancer Hospital, Baotou, China; 4 Department of Clinical Oncolygy, The Affiliated Hospital of Qingdao University, Qingdao, China; 5 Department of Medical Oncology, Chaoyang Second Hospital, Chaoyang, China; 6 Department of Cancer Center, Union Hospital, Tongji Medical College, Huazhong University of Science and Technology, Wuhan, China; 7 Department of Oncology Department, Jinzhou Medical University, Jinzhou, China; 8 Department of Respiratory and Critical Care, Chifeng Municipal Hospital, Chifeng, Inner Mongolia China; 9 Department of Radiation Oncology, Tianjin Medical University Cancer Institute & Hospital, Tianjin, China; 10 Department of Radiation Oncology, Qilu Hospital of Shandong University, Jinan, China; 11 Department of Radiology, Shandong Provincial Hospital, Jinan, China; 12 Department of Oncology, Zibo Municipal Hospital, Zibo, China

**Keywords:** Extensive stage small cell lung cancer, PD-L1 inhibitors, Thoracic radiotherapy, Survival, Toxicity

## Abstract

**Background:**

The CREST study showed that the addition of thoracic radiotherapy (TRT) could improve the survival rate in patients with extensive stage small cell lung cancer (ES-SCLC), but whether TRT can bring survival benefit in the era of immunotherapy remains controversial. This study aimed to explore the efficacy and safety of adding TRT to the combination of PD-L1 inhibitors and chemotherapy.

**Methods:**

The patients who received durvalumab or atezolizumab combined with chemotherapy as the first-line treatment of ES-SCLC from January 2019 to December 2021 were enrolled. They were divided into two groups, based on whether they received TRT or not. Propensity score matching (PSM) with a 1:1 ratio was performed. The primary endpoints were progression-free survival (PFS), overall survival (OS) and safety.

**Results:**

A total of 211 patients with ES-SCLC were enrolled, of whom 70 (33.2%) patients received standard therapy plus TRT as first-line treatment, and 141 (66.8%) patients in the control group received PD-L1 inhibitors plus chemotherapy. After PSM, a total of 57 pairs of patients were enrolled in the analysis. In all patients, the median PFS (mPFS) in the TRT and non-TRT group was 9.5 and 7.2 months, respectively, with HR = 0.59 (95%CI 0.39–0.88, *p* = 0.009). The median OS (mOS) in the TRT group was also significantly longer than that in the non-TRT group (24.1 months vs. 18.5 months, HR = 0.53, 95%CI 0.31–0.89, *p* = 0.016). Multivariable analysis showed that baseline liver metastasis and the number of metastases ≥ 3 were independent prognostic factors for OS. Addition of TRT increased the incidence of treatment-related pneumonia (*p* = 0.018), most of which were grade 1–2.

**Conclusions:**

Addition of TRT to durvalumab or atezolizumab plus chemotherapy significantly improves survival in ES-SCLC. Although it may leads to increased incidence of treatment-related pneumonia, a majority of the cases can be relieved after symptomatic treatment.

## Introduction

Small cell lung cancer (SCLC) accounts for approximately 15% of all newly diagnosed lung cancers and is highly aggressive, with approximately two-thirds of patients having advanced disease progression, known as extensive stage SCLC (ES-SCLC), at the time of diagnosis [1]. In the pre-immunotherapy era, thoracic radiotherapy (TRT) plays an important role in the treatment of ES-SCLC. CREST study showed that for patients with ES-SCLC who had a response to chemotherapy (CR/PR), radiotherapy to the primary thoracic tumor (30 Gy/10f) combined with prophylactic cranial irradiation (PCI) reduced the risk of thoracic recurrence by 50% and improved the 2-year overall survival rate (13% vs. 3%, *p* = 0.004) [2]. Therefore, the Chinese Society of Clinical Oncology (CSCO) recommend TRT for patients with ES-SCLC with CR/PR after first-line treatment and good physical conditions. In addition, the American College of Radiology (ASTRO) clinical practice guidelines also strongly recommend TRT for patients with ES-SCLC after chemotherapy [3].

In recent years, the emergence of PD-L1 inhibitors has improved the survival of ES-SCLC. Based on the CASPIAN and IMpower133 trials, two PD-L1 inhibitors, duvalumab and atezolizumab, combined with chemotherapy, have been recommended as the new standard first-line treatment of ES-SCLC by the guidelines [4, 5]. However, the median overall survival (mOS) is 12–13 months, only 2–3 months higher than that of the control group, which is still far from meeting the clinical needs. Therefore, new treatment modalities are urgently required to further improve the overall survival of patients with ES-SCLC.

Whether to receive TRT for ES-SCLC remains controversial in the immunotherapy era. Some researchers believe that immunotherapy combined with chemotherapy activates the immune system, and the addition of TRT may kill the activated immune cells [6]. In addition, immunotherapy combined with TRT may increase the incidence of interstitial pneumonia, thus delaying immunotherapy and affecting the systemic therapeutic effect [7]. The opposing view is that progression-free survival (PFS) of ES-SCLC in the immunotherapy era is not ideal, and the thorax is still the main site of progression. Approximately 75% of patients with ES-SCLC exhibit persistent intrathoracic diseases after first-line chemotherapy, and the control of intrathoracic diseases is of great significance for delaying disease progression and realizing long-term survival [8, 9]. Therefore, on the basis of PD-L1 inhibitors combined with chemotherapy, whether TRT can further improve the efficacy of patients with ES-SCLC has an emerging research topic; however, effective clinical evidence is still lacking. This study aimed to explore the efficacy and safety of TRT combined with the current standard treatment mode (PD-L1 inhibitors combined with chemotherapy) based on real-world data.

## Methods

### Study design and patients

This real-world study enrolled patients who were treated at 12 sites in China between January 2019 and December 2021. Adult patients (≥ 18 years of age) with ES-SCLC, confirmed histologically or cytologically, were enrolled in the analysis. The baseline characteristics and clinical data of the two groups were collected. The collected information included age, sex, smoking status, Eastern Cooperative Oncology Group performance status (ECOG PS), tumor stage, immunotherapy and chemotherapy regimens, baseline metastasis, thoracic radiotherapy, and survival status. At least one lesion can be assessed according to Response Evaluation Criteria in Solid Tumors (RECIST) version 1.1. The PFS and overall survival (OS) of the patients were statistically analyzed, and the statistical differences between the two groups were compared. The predictive factors affecting the efficacy were further analyzed.

### Treatment and response evaluation

All patients received PD-L1 inhibitors (duvalumab and atezolizumab) plus platinum-based chemotherapy as first-line treatment for ES-SCLC. All drugs were administered intravenously. All patients who enrolled were divided into two groups according to whether the primary tumor received TRT or not. One group received TRT in addition to PD-L1 inhibitors plus chemotherapy. The other group received PD-L1 inhibitors plus chemotherapy alone, followed by immune-maintenance therapy. Treatment was initiated in 3-week cycles followed by maintenance infusions of either duvalumab or atezolizumab every 3 weeks until disease progression, death, or unacceptable toxicity, as assessed according to RECIST, version 1.1. Initial imaging assessments including cervical, chest and abdomen contrast-enhanced computed tomography (CT), ultrasound examination, brain contrast-enhanced MRI or CT were performed every 6 weeks and then every 6–12 weeks during the maintenance phase. Positron emission tomography (PET)-CT and emission computed tomograph (ECT) was used in some patients prior to systemic therapy or radiotherapy (not routinely used).

### Outcomes

The primary endpoints were PFS, OS, and safety. PFS was defined as the time from the first day of chemotherapy to radiographic confirmation of disease progression or death, according to RECIST, version 1.1. OS was defined as the time from the first day of chemotherapy to the date of death from any cause or the last follow-up visit. The secondary endpoints were 12- and 18-month survival rates.

### Toxicity assessment

With respect to adverse events, we focused on the occurrence of treatment-related pneumonia in the two groups. The differential diagnosis of checkpoint inhibitor pneumonitis (CIP) and radiation pneumonitis (RP) is mainly based on the onset time of pneumonia, the relationship between the extent of pneumonia and the radiation range, and CT characteristics. RP mostly occurs within or at the edge of the radiation field in less than 6 months after TRT [10]. In contrast, CIP occurrs from several hours to 24 months after the first ICI treatment, with a median time of 2–3 months and a broader range of CT manifestations [11]. Pneumonitis was graded with the use of the Common Terminology Criteria for Adverse Events (version 5). Grade 1 was defined as radiographic changes confined to a single lobe or less than 25% of the lung parenchyma without any symptoms. Grade 2 is radiographic changes involving 25–50% of the lung parenchyma, with mild symptoms that do not limit daily life. Grade 3 is radiographic changes involving all lobes or more than 50% of the lung parenchyma, with severe respiratory symptoms that limit the individual's ability to perform self-care. Grade 4 is life-threatening symptoms requiring urgent intervention, and grade 5 is death.

### Statistical analysis

Propensity score matching (PSM) was performed to adjust for imbalances between the two groups, with paired covariates including age, gender, ECOG PS, weight loss, smoking history, baseline brain metastases, baseline liver metastases, number of metastases, and therapeutic regimens. Patients in the two cohorts were then matched by nearest neighbour matching in a 1:1 ratio. A calliper of 0.02 was applied as the match tolerance of the paired propensity score. Descriptive statistics using counts (percentages) for categorical variables and medians (IQR) for continuous variables were used to summarize the baseline characteristics of the patients and the distribution of treatment exposure. Chi-squared tests or Fisher’s exact tests were generated for categorical variables and Mann–Whitney tests for continuous variables. Kaplan–Meier survival curve and log-rank test were used to compare the differences in survival between the two treatment groups, and the corresponding median survival time and 12- and 18-month survival rates of the population were calculated. Multivariate analyses of survival outcomes were performed using Cox proportional-hazards models with estimated hazard ratios (HRs) and 95% confidence intervals (CIs). Data from patients who had not experienced disease progression or death at the time of analysis were censored on the day of the last available RECIST assessment. Two-tailed tests and *p* values < 0.05 for significance were implemented. All statistical analyses were performed using the SPSS (version 25.0, IBM Corporation, USA) and GraphPad Prism (version 7.0, GraphPad Software, USA).

## Results

### Patients and treatment exposure

**Fig. 1:**
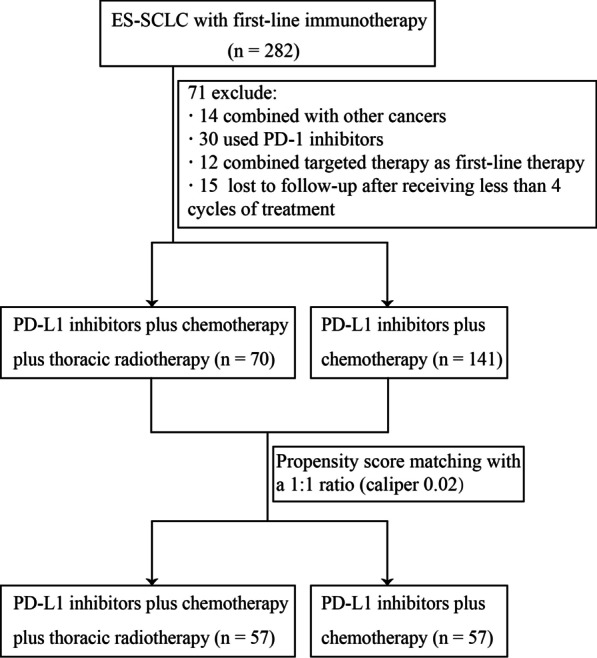
Patient selection flowchart. ES-SCLC, extensive stage small cell lung cancer; PD-1, programmed cell death 1; PD-L1, programmed cell death ligand 1

A total of 211 patients with ES-SCLC who received either atezolizumab or duvalumab combined with standard chemotherapy as first-line therapy between January 2019 and December 2021 were enrolled, of which 70 patients were additionally treated with TRT (Fig. 1). Specific baseline and treatment characteristics are shown in Table 1. Overall, a majority of patients were younger than 65 years (133, 63.0%) and were male (171, 81.0%). Most patients had ECOG PS score of 0–1 at diagnosis (184, 87.2%), and the most common PD-L1 inhibitor was duvalumab (141, 66.8%).

**Table 1:** Baseline and therapeutic characteristics of patients before and after PSM

Characteristics	Before matching			After matching		
	With TRT (n = 70) (%)	Wihout TRT (n = 141) (%)	*p* value	With TRT (n = 57) (%)	Wihout TRT (n = 57) (%)	*p* value
Age, median (IQR), yr	61 (54–67)	63 (56–68)	0.237	63 (56–69)	61 (55–66)	0.735
< 65	45 (64.3)	88 (62.4)	0.791	33 (57.9)	38 (66.7)	0.334
≥ 65	25 (35.7)	53 (37.6)		24 (42.1)	19 (33.3)	
Gender			0.078			0.826
Male	52 (74.3)	119 (84.4)		43 (75.4)	44 (77.2)	
Female	18 (25.7)	22 (15.6)		14 (24.6)	13 (22.8)	
ECOG PS			0.196			0.242
0–1	64 (91.4)	120 (85.1)		52 (91.2)	55 (96.5)	
≥ 2	6 (8.6)	21 (14.9)		5 (8.8)	2 (3.5)	
Weight loss			0.219			0.281
Yes	14 (20.0)	19 (13.5)		10 (17.5)	6 (10.5)	
No	56 (80.0)	122 (86.5)		47 (82.5)	51 (89.5)	
Smoking status			0.669			0.729
Never	27 (38.6)	46 (32.6)		21 (36.8)	17 (29.8)	
Former	21 (30.0)	44 (31.2)		19 (33.3)	21 (36.8)	
Current	22 (31.4)	51 (36.2)		17 (29.8)	19 (33.3)	
Brain metastases			0.384			0.528
Yes	15 (21.4)	38 (27.0)		14 (24.6)	17 (29.8)	
No	55 (78.6)	103 (73.0)		43 (75.4)	40 (70.2)	
Liver metastases			0.032			0.528
Yes	16 (22.9)	53 (37.6)		14 (24.6)	17 (29.8)	
No	54 (77.1)	88 (62.4)		43 (75.4)	40 (70.2)	
Number of metastases			0.026			0.192
< 3	59 (84.3)	99 (70.2)		46 (80.7)	40 (70.2)	
≥ 3	11 (15.7)	42 (29.8)		11 (19.3)	17 (29.8)	
Immunotherapy regimens			0.809			0.341
Durvalumab	46 (65.7)	95 (67.4)		36 (63.2)	31 (54.4)	
Atezolizumab	24 (34.3)	46 (32.6)		21 (36.8)	26 (45.6)	
Cycles of immunotherapy			0.031			0.687
< 6	20 (28.6)	62 (44.0)		17 (29.8)	19 (33.3)	
≥ 6	50 (71.4)	79 (56.0)		40 (70.2)	38 (66.7)	
Chemotherapy regimens			0.179			0.559
Etoposide + cisplatin	36 (51.4)	57 (40.4)		26 (45.6)	29 (50.9)	
Etoposide + carboplatin	28 (40.0)	61 (43.3)		25 (43.9)	25 (43.9)	
Others	6 (8.6)	23 (16.3)		6 (10.5)	3 (5.3)	
TRT sequence			–			–
Sequential	47 (67.1)	–		42 (73.7)	–	
Concurrent	23 (32.9)	–		15 (26.3)	–	

PSM, propensity score matching; TRT, thoracic radiotherapy; IQR, interquartile range; yr, years; ECOG PS, Eastern Cooperative Oncology Group performance status

A total of 57 pairs completed propensity score matching, and the covariates between the two groups were balanced after matching (Table 1). In the post-PSM analysis set, all patients received PD-L1 inhibitors (duvalumab and atezolizumab) plus platinum-based chemotherapy, 65.8% received concurrent chemotherapy plus immunotherapy from the first cycle, and 34.2% received combination immunotherapy during chemotherapy. The median cycles of chemotherapy were 6 in both the TRT group (IQR: 4–6) and the non-TRT group (IQR: 5–6). The median total cycles of immunotherapy (including maintenance therapy) in the TRT and non-TRT groups were 7 (IQR: 5–10) and 6 (IQR: 5–8), respectively. In the TRT group, a total of 42 patients (73.7%) received sequential TRT, with a subset of patients receiving fractional radiotherapy (3 Gy per fractional) received sequential TRT in the interval of two systemic treatments and a subset of some patients were unable to tolerate concurrent radiotherapy. Due to the low tumor burden and number of metastases in some patients, 15 patients (26.3%) received synchronous TRT within the first 2 cycles of systemic therapy based on their condition, willingness, and physician evaluation. According to the Chinese Society of Clinical Oncology (CSCO) guidelines or National Comprehensive Cancer Network (NCCN) guidelines, the patients received TRT at fractionated doses of 30 Gy/10f (19.2%) and 45 Gy/15f (21.1%) with 3 Gy per fraction, 50 Gy/25f (21.1%) and 60 Gy/30f (38.6%) with 2.0 Gy per fraction, and the median dose of TRT was 50 Gy (IQR: 45–60). TRT was performed using intensity-modulated radiotherapy. The gross target volume included residual primary lesions and positive lymph nodes after treatment, and the clinical target volume included gross target volume + 8 mm margin and nodal regions involved before. In addition, 17 patients received prophylactic cranial irradiation (PCI), and the 25 Gy/10f scheme was the most commonly used. Of the 31 patients with brain metastases, 21 were treated with cranial irradiation (11 in TRT, 10 in non-TRT). 4 patients in both groups received radiotherapy for bone metastases. One patient in the TRT group received radiotherapy for retroperitoneal lymph node metastases after systemic therapy.

### Real-world efficacy of TRT on patients

**Fig. 2:**
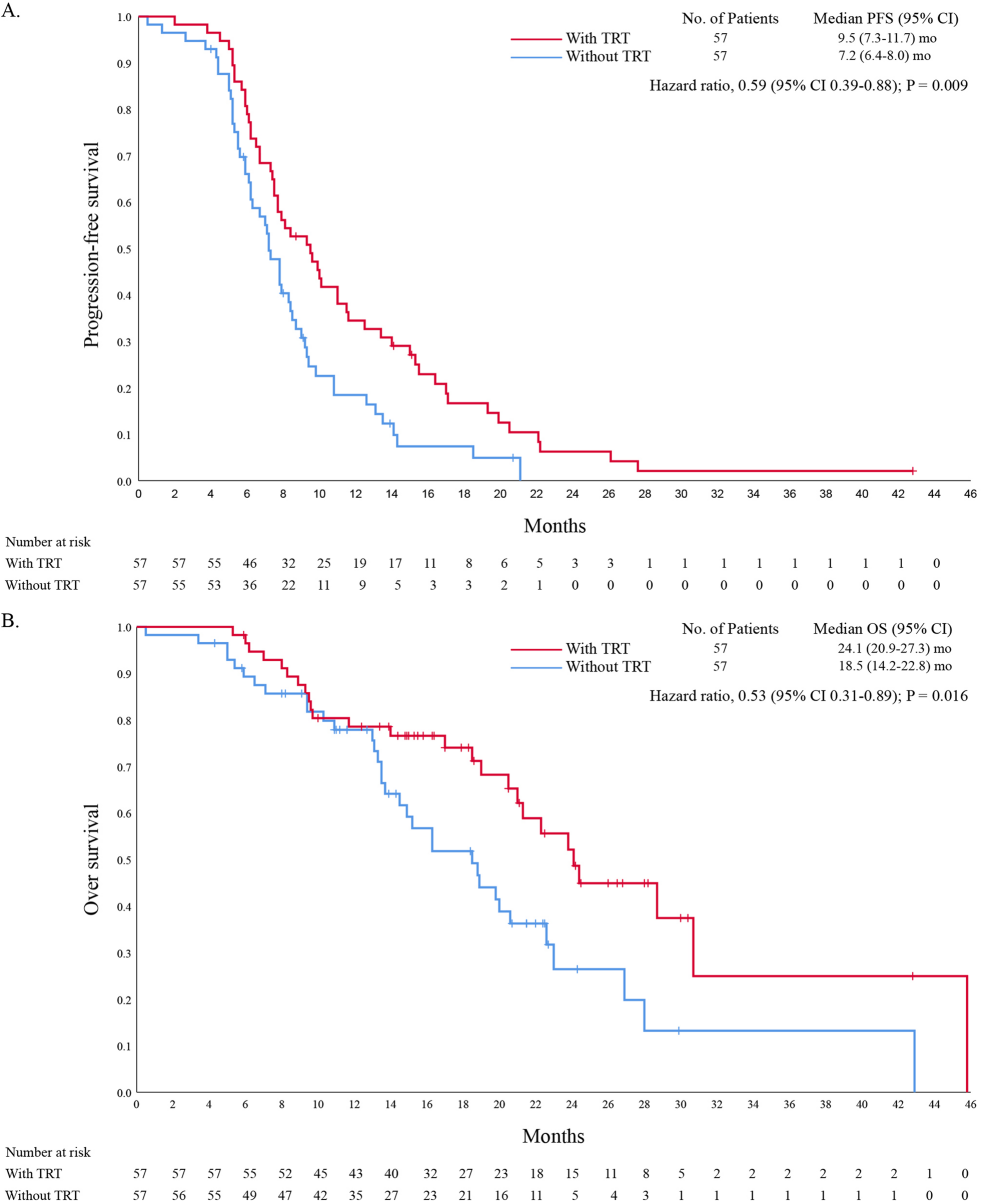
Kaplan–Meier PFS and OS for all patients. TRT, thoracic radiotherapy; PFS, progression-free survival; OS, overall survival; mo: months; CI, confidence interval

The median follow-up time was 24.2 and 22.0 months in the TRT vs. non-TRT cohorts, respectively. As of November 2022, 53 (93.0%) patients in TRT group and 51 (89.5%) patients in the TRT and non-TRT groups experienced disease progression or died (*p* = 0.508), respectively, as defined by RECIST, version 1.1. PFS was significantly longer in patients who received TRT than in those who did not, with mPFS of 9.5 months and 7.2 months, respectively, HR = 0.59 (95%CI 0.39–0.88, *p* = 0.009). The 6-month PFS rates were 80.0% (95% CI 65.8–88.2) vs. 61.4% (95% CI 47.6–73.7) (*p* = 0.041); the 12-month PFS rates were 31.6% (95% CI 20.3–45.4) and 14.0% (95% CI 6.7–26.4) (*p* = 0.026), respectively (Fig. 2A).

**Fig. 3:**
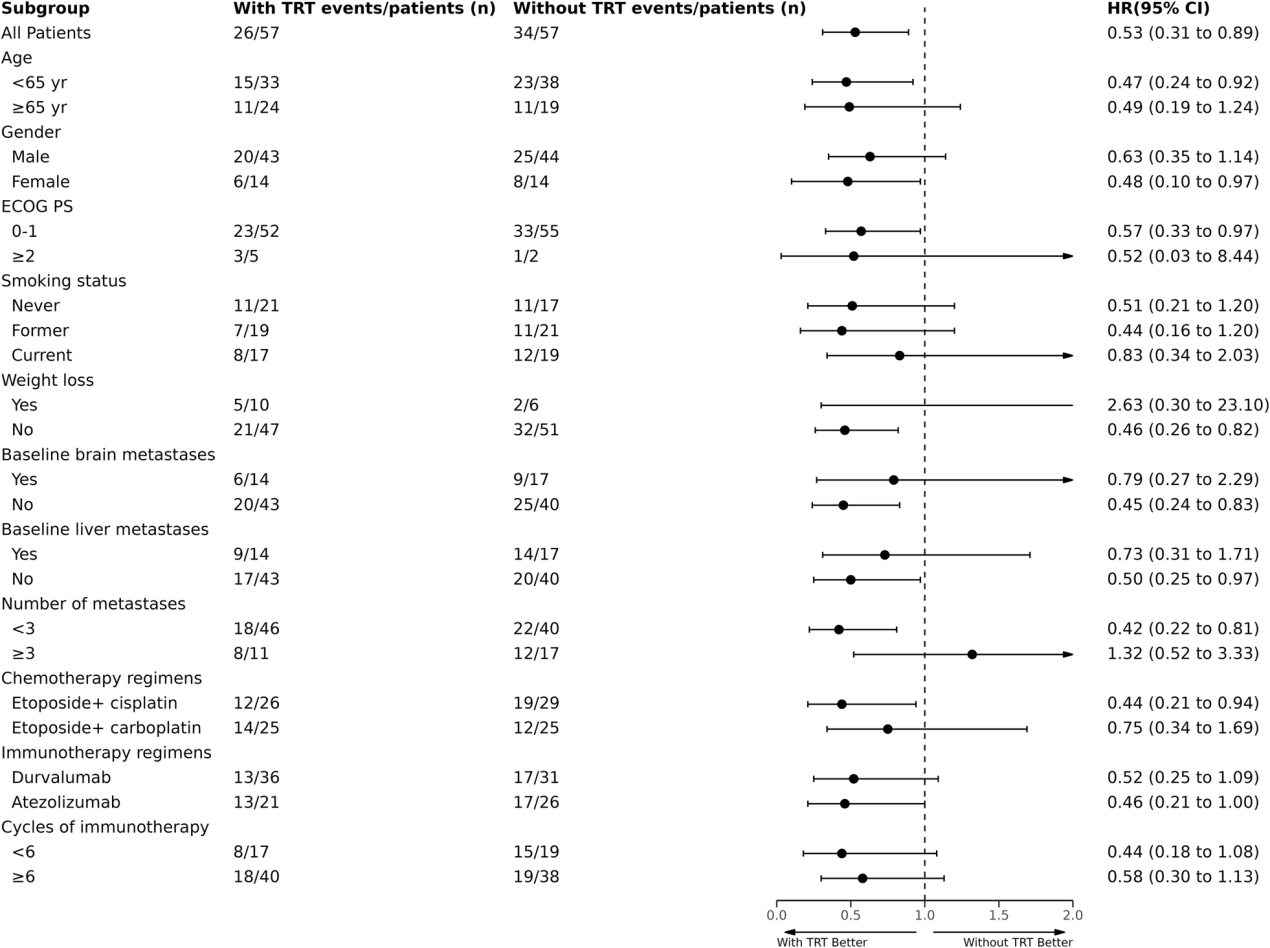
Forest plot of subgroup analysis of OS. OS, overall survival; TRT, thoracic radiotherapy; ECOG PS, Eastern Cooperative Oncology Group performance status; HR, hazard ratio; CI, confidence interval

By the end of follow-up, death had occurred in 26 (45.6%) and 34 (59.6%) patients in the TRT and non-TRT groups, respectively. OS at 12 month was 73.7% (95% CI 60.1–84.1) and 59.7% (95% CI 45.8–72.2) in TRT and non-TRT groups (*p* = 0.072), respectively. At 18 months, survival was 45.6% (95% CI 32.6–59.2) vs. 35.1% (95% CI 23.3–48.9) (*p* = 0.252), respectively. Median OS was longer in the TRT plus PD-L1 inhibitors plus chemotheropy cohort compared with PD-L1 inhibitors plus chemotheropy cohort (24.1 months: 18.5 months; HR = 0.53, 95% CI 0.31–0.89, *p* = 0.016; Fig. 2B). In exploratory subgroup analyses of OS (Fig. 3), significant differences in OS were observed among groups including female, ECOG score of 0–1, without weight loss, no baseline brain or liver metastases, number of metastases < 3 and etoposide plus cisplatin.

### Prognostic factors for OS

**Fig. 4:**
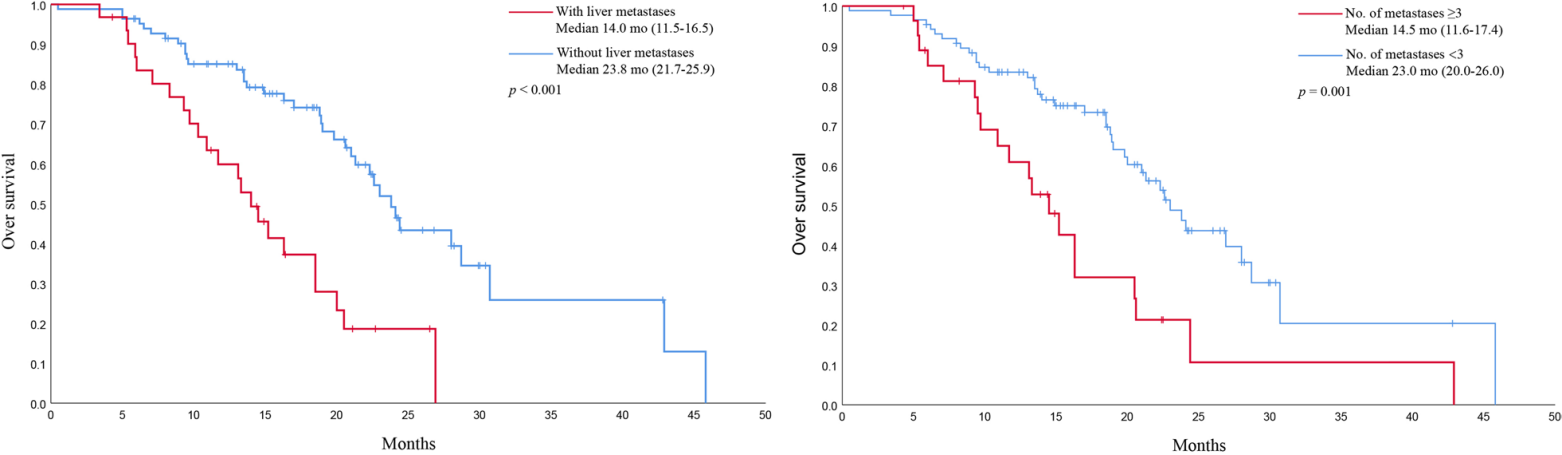
Kaplan–Meier OS for patients with risk factors. OS, overall survival; mo, months

We evaluated prognostic factors for OS using univariate and multivariate Cox proportional hazards regression analyses (Table 2). The final multivariate cox model analysis showed that baseline liver metastasis (HR = 2.58, 95% CI 1.45–4.58) and the number of metastases ≥ 3 (HR = 1.86, 95% CI 1.04–3.32) were risk factors for poor OS. Survival curves for OS were subsequently plotted according to risk factors, and the results showed that patients with baseline liver (14.0 months vs. 23.8 months, *p* < 0.001) and the number of metastases ≥ 3 (14.5 months vs. 23.0 months, *p* = 0.009) had significantly shorter OS compared with those without (Fig. 4). No statistical survival benefit was observed in patients who underwent further PCI.

**Table 2:** Univariate and multivariable analyses for OS

Characteristics	Univariate analysis		Multivariable analysis	
	HR (95% CI)	*p* value	HR (95% CI)	*p* value
Age				
(< 65 yr vs. ≥ 65 yr)	1.13 (0.66–1.92)	0.660	–	0.171
Gender				
(Male vs. Female)	1.22 (0.65–2.28)	0.542	–	0.961
ECOG PS				
(0–1 vs. ≥ 2)	0.43 (0.13–1.41)	0.166	–	0.198
Smoking history				
(Yes vs. No)	1.27 (0.73–2.23)	0.385	–	0.593
Weight loss				
(Yes vs. No)	0.66 (0.30–1.47)	0.308	–	0.215
Baseline brain metastases				
(Yes vs. No)	0.95 (0.53–1.72)	0.873	–	0.317
Baseline liver metastases				
(Yes vs. No)	3.01 (1.75–5.20)	< 0.001	2.58 (1.45–4.58)	**0.001**
Baseline bone metastases				
(Yes vs. No)	1.81 (1.05–3.11)	0.032	–	0.069
Number of metastases				
(< 3 vs. ≥ 3)	2.39 (1.38–4.14)	0.002	1.86 (1.04–3.32)	**0.036**
Immunotherapy regimens				
(Durvalumab vs. Atezolizumab)	1.29 (0.77–2.17)	0.333	–	0.149
Cycles of immunotherapy				
(< 6 vs. ≥ 6)	0.75 (0.44–1.28)	0.287	–	0.424
PCI				
(Yes vs. No)	0.67 (0.29–1.57)	0.460	–	0.944

OS, overall survival; HR, hazard ratio; CI, confidence interval; yr, years; ECOG PS, Eastern Cooperative Oncology Group performance status; PCI, prophylactic cranial irradiation

The *p* values of the bold were ≤ 0.05, which were considered statistically significant and were used for subsequent prognostic analysis

### Treatment-related pneumonitis

**Fig. 5:**
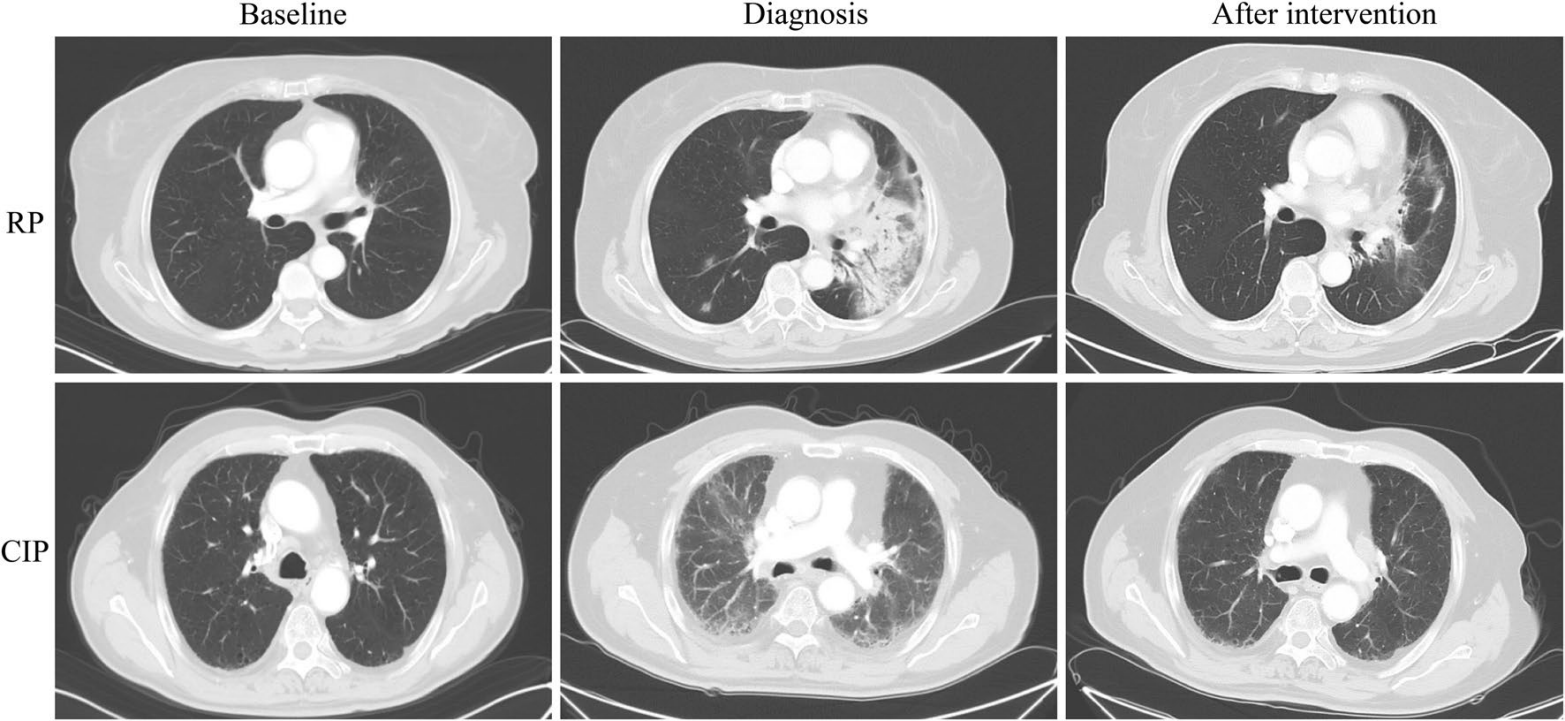
Representative CT changes of patients with RP and CIP. RP: radiation pneumonitis; CIP: checkpoint inhibitor pneumonitis

At the time of data cutoff, clinically significant treatment-related pneumonitis had occurred in 20 (35.1%) patients and 9 (15.8%) patients in the TRT and non-TRT groups (*p* = 0.018), respectively, including one death due to CIP. In the TRT group, 15 (26.3%)patients developed RP, including 5 cases of grade 1 and 7 cases of grade 2. The other 3 patients discontinued antineoplastic therapy because of grade 3–4 pneumonitis, which were improved after active steroid symptomatic treatment, expectorant and asthma treatment. No patient exhibited grade 5 RP. The CT changes of representative patients diagnosed with RP and CIP are shown in Fig. 5.

## Discussion

Chemotherapy, especially four to six cycles of platinum-based chemotherapy with etoposide plus cisplatin or carboplatin, has long been the standard of care for patients with small cell lung cancer. Although the objective effective rate of chemotherapy may be as high as 50–70%, the long-term efficacy is still poor and the 5-year survival rate is less than 7% due to the rapid progression after treatment resistance [8, 12]. In recent years, the most promising development in the treatment of ES-SCLC was the addition of immunotherapy to standard platinum-based first-line chemotherapy based on the CASPIAN and IMpower133 trials; however, neither study allowed consolidation TRT. Although both immunotherapy and TRT could individually benefit patients with ES-SCLC, evidence supporting the use of TRT in patients with ES-SCLC remains insufficient, and the optimal dose and timing of radiotherapy remain unclear. This real-world study was designed to examine the effect of addition of TRT to ES-SCLC in the era of immunotherapy. The results showed that TRT addition to standard first-line therapy (PD-L1 inhibitors plus chemotherapy) further improved PFS and OS. Baseline liver metastasis and the number of metastases ≥ 3 were independent risk predictors for OS.

Evidence from pre-clinical trials suggests that synergies may exist between immunotherapy and radiotherapy [13, 14]. Radiotherapy can remodel the tumor immune microenvironment, convert “cold” into “hot” tumors, thereby increasing the sensitivity of immunotherapy, and even improve the chances of an abscopal response. First, radiotherapy induces immunogenic cell death and promotes neoantigen accumulation in tumors. Second, radiotherapy can induce the up-regulation of MHC-I expression on the surface of tumor cells, and can activate the proliferation and activation of dendritic cells and T cells through a variety of pathways. Third, radiotherapy induces vascular normalization and improves T-cell homing and invasion to the tumor. In pre-clinical studies, radiotherapy combined with PD-1 [15–17], PD-L1 [18–20], and CTLA-4 [21, 22] inhibitors improved long-term survival and prevented tumor recurrence under a variety of fractionated doses.

Clinical trials involving patients with NSCLC have provided encouraging evidence regarding the survival and safety of immunotherapy combined with TRT. In the PACIFIC trial, consolidated duvalumab significantly prolonged OS compared with placebo, in patients with stage III unresectable NSCLC after concurrent chemoradiotherapy [23, 24]. The PEMBRO-RT trial showed that pembrolizumab combined with TRT significantly prolonged mPFS (9.0 months vs. 4.4 months, *p* = 0.045) and mOS (19.2 months vs. 8.7 months, *p* = 0.0004) of patients with metastatic NSCLC, compared with pembrolizumab alone, and significantly improved the rate of distant response to treatment (41.7% vs. 19.7%, *p* = 0.0039) [25]. However, there are few clinical trials on TRT combined with immunotherapy for ES-SCLC. The study by Welsh et al., which examined pembrolizumab in combination with TRT in patients with ES-SCLC after induction chemotherapy, showed that concurrent administration of pembrolizumab and TRT was well tolerated, with no grade 4–5 adverse events observed, and only 6% of patients experiencing grade 3 adverse events [26]. However, because of the heterogeneity in inclusion criteria, deriving definitive estimates of survival is difficult [26]. Similarly, the study by Diamond et al. enrolled 20 patients with ES-SCLC who received first-line chemotherapy and immunotherapy followed by TRT, with a median follow-up time of 12 months; the results showed that mOS was 16 months, the 6-month OS rate was 94.7%, and the 12-month OS rate was 77.5%, with a favorable safety profile [27]. Another Phase I trial of Ipilimumab and Nivolumab in combination with TRT after chemotherapy in ES-SCLC did not report a new toxic profile, but increased incidence of severe toxicity, with 61.9% of patients having treatment-related grade 3 or higher adverse events, and did not prolong PFS [28]. The optimal dose and timing of TRT is unknown, but current NCCN guidelines recommend that consolidative TRT after immunotherapy combined with chemotherapy may be considered during or before maintenance immunotherapy in selected patients [29].

Notably, the optimal treatment consolidation TRT is worthy of further discussion. Based on limited clinical data on RT-immunotherapy for ES-SCLC, the optimal TRT dose remains controversial. In the absence of immunotherapy, a retrospective study found that TRT greater than 50 Gy improved OS in patients with ES-SCLC compared with low doses [30]. Conversely, Han et al. [31] and Luan et al. [32] indicated that TRT at 45 Gy/30f had a better survival benefit than TRT at 60 Gy/30f. In an Italian prospective study named TRENDS, the efficacy of receiving consolidated TRT following first-line chemotherapy was evaluated. In this study, 55% of patients received TRT at 30 Gy/10f and 25% received 45 Gy/15f, and the risk of thoracic lesions progressing was reduced [33]. In the era of immunotherapy, high-dose TRT may lead to better local control and OS in ES-SCLC, while simultaneously increasing additional pulmonary toxicity. Therefore, more clinical studies should be conducted to prudently determine the most effective and feasible radiotherapy regimen.

Treatment-related pneumonitis, the most common high-grade adverse event, was most prominent with combined ICIs and TRT [7]. The formation of treatment-related pneumonia is a complex process that is co-existed, interacted, and comprehensively regulated by many factors, which is mainly attributed to excessive immune response and cytokine secretion[34–36]. ICIs in combination with TRT, either concurrently or sequentially, may lead to increased pulmonary toxicity. The reported incidences of grade 2 or higher lung injury with TRT alone or combined chemotherapy ranges from 5 to 30% [10, 37, 38], and the incidences of ICI-induced CIP ranges from 3 to –5% [39–41]. In clinical trials of ICIs after conventional TRT, the incidences of any grade pneumonia and grade ≥ 3 pneumonia were 13–33% and 1–9%, respectively, compared with 56–62% and 2% observed in the real world [11]. In a previous study from our group, we observed a significant increase in the incidence and severity of treatment-related pneumonia in patients who underwent TRT after ICIs (grade ≥ 2, 48.96%; grade ≥ 3, 19.79%), and fatal pneumonia occurred in 6.25% of patients [42]. As the primary end point of this study, we observed that the incidence of all grades, grade 2 or higher, and grade 3 or higher pneumonia in the TRT group was 35.1%, 24.6%, and 5.2%, respectively, which was higher than that in the non-TRT group; however, a majority of patients recovered from pneumonia or remained stable. This result is consistent with those of previous studies, suggesting that this new regimen may be well tolerated in ES-SCLC.

This study has several limitations. First, due to the real-world nature of the study, the patient population was heterogeneous, with selection bias. However, we attempted to control for some potential confounders by PSM to minimize the bias of retrospective type selection. In addition, the diagnosis of pneumonia is relatively subjective, and it is difficult to distinguish immune-related pneumonia from radiation pneumonitis, and the intervention measures are similar. A few patients who were lost to follow-up could not be evaluated for radiation recall pneumonitis (RRP) that occurred more than 6 months after ICI and TRT [43]. We made our best effort to evaluate pneumonia on the basis of symptoms and imageological examination in each patient.

In summary, TRT can improve the prognosis of patients with ES-SCLC receiving first-line PD-L1 inhibitors combined with chemotherapy. Few high-grade adverse events occurred in the patients who were followed up, and most of them were mild clinically manageable pneumonia. Further prospective studies in larger and more homogeneous patients are needed to validate these results.

## Data Availability

The datasets used and/or analysed during the current study are available from the corresponding author on reasonable request.

## Abbreviations

ES-SCLC: Extensive stage small cell lung cancer
TRT: Thoracic radiotherapy
OS: Overall survival
PFS: Progression-free survival
PSM: Propensity score matching
mPFS: Median progression-free survival
mOS: Median overall survival
NSCLC: Non-small cell lung cancer
SoC: Standard of care
PCI: Prophylactic cranial irradiation
CSCO: The Chinese society of clinical oncology
ASTRO: The American college of radiology
PD-1: Programmed cell death 1
PD-L1: Programmed cell death ligand 1
CTLA-4: Cytotoxic T lymphocyte-associated antigen 4
ECOG PS: Eastern cooperative oncology group performance status
RECIST: Response evaluation criteria in solid tumors
CR: Complete response
PR: Partial response
CIP: Checkpoint inhibitor pneumonitis
RP: Radiation pneumonitis
HRs: Hazard ratios
CIs: Confidence intervals
RRP: Radiation recall pneumonitis

## References

[1] Rudin CM, Brambilla E, Faivre-Finn C, Sage J (2021). Small-cell lung cancer. Nat Rev Dis Primers.

[2] Slotman BJ, van Tinteren H, Praag JO, Knegjens JL, El Sharouni SY, Hatton M, Keijser A, Faivre-Finn C, Senan S (2015). Use of thoracic radiotherapy for extensive stage small-cell lung cancer: a phase 3 randomised controlled trial. Lancet.

[3] Simone CB, Bogart JA, Cabrera AR, Daly ME, DeNunzio NJ, Detterbeck F, Faivre-Finn C, Gatschet N, Gore E, Jabbour SK, Kruser TJ, Schneider BJ, Slotman B, Turrisi A, Wu AJ, Zeng J, Rosenzweig KE (2020). Radiation therapy for small cell lung cancer: an ASTRO clinical practice guideline. Pract Radiat Oncol.

[4] Horn L, Mansfield AS, Szczęsna A, Havel L, Krzakowski M, Hochmair MJ, Huemer F, Losonczy G, Johnson ML, Nishio M, Reck M, Mok T, Lam S, Shames DS, Liu J, Ding B, Lopez-Chavez A, Kabbinavar F, Lin W, Sandler A, Liu SV (2018). First-line atezolizumab plus chemotherapy in extensive-stage small-cell lung cancer. N Engl J Med.

[5] Paz-Ares L, Dvorkin M, Chen Y, Reinmuth N, Hotta K, Trukhin D, Statsenko G, Hochmair MJ, Özgüroğlu M, Ji JH, Voitko O, Poltoratskiy A, Ponce S, Verderame F, Havel L, Bondarenko I, Kazarnowicz A, Losonczy G, Conev NV, Armstrong J, Byrne N, Shire N, Jiang H, Goldman JW (2019). Durvalumab plus platinum-etoposide versus platinum-etoposide in first-line treatment of extensive-stage small-cell lung cancer (CASPIAN): a randomised, controlled, open-label, phase 3 trial. Lancet.

[6] Li H, Yu H, Lan S, Zhao D, Liu Y, Cheng Y (2021). Aberrant alteration of circulating lymphocyte subsets in small cell lung cancer patients treated with radiotherapy. Technol Cancer Res Treat.

[7] Li B, Jiang C, Pang L, Zou B, Ding M, Sun X, Yu J, Wang L (2021). Toxicity profile of combining PD-1/PD-L1 inhibitors and thoracic radiotherapy in non-small cell lung cancer: a systematic review. Front Immunol.

[8] Tian Y, Ma J, Jing X, Zhai X, Li Y, Guo Z, Yu J, Zhu H (2022). Radiation therapy for extensive-stage small-cell lung cancer in the era of immunotherapy. Cancer Lett.

[9] Guaitoli G, Neri G, Cabitza E, Natalizio S, Mastrodomenico L, Talerico S, Trudu L, Lauro C, Chiavelli C, Baschieri MC, Bruni A, Dominici M, Bertolini F (2022). Dissecting immunotherapy strategies for small cell lung cancer: antibodies, ionizing radiation and CAR-T. Int J Mol Sci.

[10] Hanania AN, Mainwaring W, Ghebre YT, Hanania NA, Ludwig M (2019). Radiation-induced lung injury: assessment and management. Chest.

[11] Lu X, Wang J, Zhang T, Zhou Z, Deng L, Wang X, Wang W, Liu W, Tang W, Wang Z, Wang J, Jiang W, Bi N, Wang L (2022). Comprehensive pneumonitis profile of thoracic radiotherapy followed by immune checkpoint inhibitor and risk factors for radiation recall pneumonitis in lung cancer. Front Immunol.

[12] Byers LA, Rudin CM (2015). Small cell lung cancer: where do we go from here?. Cancer.

[13] Formenti SC, Demaria S (2013). Combining radiotherapy and cancer immunotherapy: a paradigm shift. J Natl Cancer Inst.

[14] Demaria S, Golden EB, Formenti SC (2015). Role of local radiation therapy in cancer immunotherapy. JAMA Oncol.

[15] Zeng J, See AP, Phallen J, Jackson CM, Belcaid Z, Ruzevick J, Durham N, Meyer C, Harris TJ, Albesiano E, Pradilla G, Ford E, Wong J, Hammers HJ, Mathios D, Tyler B, Brem H, Tran PT, Pardoll D, Drake CG, Lim M (2013). Anti-PD-1 blockade and stereotactic radiation produce long-term survival in mice with intracranial gliomas. Int J Radiat Oncol Biol Phys.

[16] Park SS, Dong H, Liu X, Harrington SM, Krco CJ, Grams MP, Mansfield AS, Furutani KM, Olivier KR, Kwon ED (2015). PD-1 restrains radiotherapy-induced abscopal effect. Cancer Immunol Res.

[17] Dovedi SJ, Cheadle EJ, Popple AL, Poon E, Morrow M, Stewart R, Yusko EC, Sanders CM, Vignali M, Emerson RO, Robins HS, Wilkinson RW, Honeychurch J, Illidge TM (2017). Fractionated radiation therapy stimulates antitumor immunity mediated by both resident and infiltrating polyclonal T-cell populations when combined with PD-1 blockade. Clin Can Res Off J Am Assoc Can Res.

[18] Dudzinski SO, Cameron BD, Wang J, Rathmell JC, Giorgio TD, Kirschner AN (2019). Combination immunotherapy and radiotherapy causes an abscopal treatment response in a mouse model of castration resistant prostate cancer. J Immunother Cancer.

[19] Deng L, Liang H, Burnette B, Beckett M, Darga T, Weichselbaum RR, Fu YX (2014). Irradiation and anti-PD-L1 treatment synergistically promote antitumor immunity in mice. J Clin Investig.

[20] Philippou Y, Sjoberg HT, Murphy E, Alyacoubi S, Jones KI, Gordon-Weeks AN, Phyu S, Parkes EE, Gillies McKenna W, Lamb AD, Gileadi U, Cerundolo V, Scheiblin DA, Lockett SJ, Wink DA, Mills IG, Hamdy FC, Muschel RJ, Bryant RJ (2020). Impacts of combining anti-PD-L1 immunotherapy and radiotherapy on the tumour immune microenvironment in a murine prostate cancer model. British J Can.

[21] Yoshimoto Y, Suzuki Y, Mimura K, Ando K, Oike T, Sato H, Okonogi N, Maruyama T, Izawa S, Noda SE, Fujii H, Kono K, Nakano T (2014). Radiotherapy-induced anti-tumor immunity contributes to the therapeutic efficacy of irradiation and can be augmented by CTLA-4 blockade in a mouse model. PLoS ONE.

[22] Dewan MZ, Galloway AE, Kawashima N, Dewyngaert JK, Babb JS, Formenti SC, Demaria S (2009). Fractionated but not single-dose radiotherapy induces an immune-mediated abscopal effect when combined with anti-CTLA-4 antibody. Clin Cancer Res Off J Am Assoc Can Res.

[23] Antonia SJ, Villegas A, Daniel D, Vicente D, Murakami S, Hui R, Yokoi T, Chiappori A, Lee KH, de Wit M, Cho BC, Bourhaba M, Quantin X, Tokito T, Mekhail T, Planchard D, Kim YC, Karapetis CS, Hiret S, Ostoros G, Kubota K, Gray JE, Paz-Ares L, de Castro Carpeño J, Wadsworth C, Melillo G, Jiang H, Huang Y, Dennis PA, Özgüroğlu M (2017). Durvalumab after chemoradiotherapy in stage III non-small-cell lung cancer. N Engl J Med.

[24] Spigel DR, Faivre-Finn C, Gray JE, Vicente D, Planchard D, Paz-Ares L, Vansteenkiste JF, Garassino MC, Hui R, Quantin X, Rimner A, Wu YL, Özgüroğlu M, Lee KH, Kato T, de Wit M, Kurata T, Reck M, Cho BC, Senan S, Naidoo J, Mann H, Newton M, Thiyagarajah P, Antonia SJ (2022). Five-year survival outcomes from the PACIFIC trial: durvalumab after chemoradiotherapy in stage III Non-Small-Cell Lung Cancer. J Clin Oncol Off J Am Soc Clin Oncol.

[25] Theelen W, Peulen HMU, Lalezari F, van der Noort V, de Vries JF, Aerts J, Dumoulin DW, Bahce I, Niemeijer AN, de Langen AJ, Monkhorst K, Baas P (2019). Effect of pembrolizumab after stereotactic body radiotherapy vs pembrolizumab alone on tumor response in patients with advanced non-small cell lung cancer: results of the PEMBRO-RT Phase 2 randomized clinical trial. JAMA Oncol.

[26] Welsh JW, Heymach JV, Chen D, Verma V, Cushman TR, Hess KR, Shroff G, Tang C, Skoulidis F, Jeter M, Menon H, Nguyen QN, Chang JY, Altan M, Papadimitrakopoulou VA, Simon GR, Raju U, Byers L, Glisson B (2020). Phase I trial of pembrolizumab and radiation therapy after induction chemotherapy for extensive-stage small cell lung cancer. J Thoracic Oncol Off Publ Int Assoc Stud Lung Can.

[27] Diamond BH, Verma N, Shukla UC, Park HS, Koffer PP (2022). Consolidative thoracic radiation therapy after first-line chemotherapy and immunotherapy in extensive-stage small cell lung cancer: a multi-institutional case series. Adv Radiat Oncol.

[28] Perez BA, Kim S, Wang M, Karimi AM, Powell C, Li J, Dilling TJ, Chiappori A, Latifi K, Rose T, Lannon A, MacMillan G, Saller J, Grass GD, Rosenberg S, Gray J, Haura E, Creelan B, Tanvetyanon T, Saltos A, Shafique M, Boyle TA, Schell MJ, Conejo-Garcia JR, Antonia SJ (2021). Prospective single-arm phase 1 and 2 study: Ipilimumab and Nivolumab with thoracic radiation therapy after platinum chemotherapy in extensive-stage small cell lung cancer. Int J Radiat Oncol Biol Phys.

[29] Ganti AKP, Loo BW, Bassetti M, Blakely C, Chiang A, D'Amico TA, D'Avella C, Dowlati A, Downey RJ, Edelman M, Florsheim C, Gold KA, Goldman JW, Grecula JC, Hann C, Iams W, Iyengar P, Kelly K, Khalil M, Koczywas M, Merritt RE, Mohindra N, Molina J, Moran C, Pokharel S, Puri S, Qin A, Rusthoven C, Sands J, Santana-Davila R, Shafique M, Waqar SN, Gregory KM, Hughes M (2021). Small cell lung cancer, version 2.2022, NCCN clinical practice guidelines in oncology. J Natl Compr Can Netw JNCCN.

[30] Li-Ming X, Zhao LJ, Simone CB, Cheng C, Kang M, Wang X, Gong LL, Pang QS, Wang J, Yuan ZY, Wang P (2017). Receipt of thoracic radiation therapy and radiotherapy dose are correlated with outcomes in a retrospective study of three hundred and six patients with extensive stage small-cell lung cancer. Radiother Oncol J European Soc Ther Radiol Oncol.

[31] Han J, Fu C, Li B (2021). Clinical outcomes of extensive-stage small cell lung cancer patients treated with thoracic radiotherapy at different times and fractionations. Radiation Oncol.

[32] Luan Z, Wang Z, Huang W, Zhang J, Dong W, Zhang W, Li B, Zhou T, Li H, Zhang Z, Wang Z, Sun H, Yi Y (2015). Efficacy of 3D conformal thoracic radiotherapy for extensive-stage small-cell lung cancer: a retrospective study. Exp Ther Med.

[33] Cozzi S, Bruni A, Ruggieri MP, Borghetti P, Scotti V, Franceschini D, Fiore M, Taraborrelli M, Salvi F, Galaverni M, Savoldi L, Braglia L, Botti A, Finocchi Ghersi S, Niccolò GL, Lohr F, Iotti C, Ciammella P (2023). Thoracic radiotherapy in extensive disease small cell lung cancer: multicenter prospective observational TRENDS study. Cancers.

[34] Yin J, Wu Y, Yang X, Gan L, Xue J (2022). Checkpoint inhibitor pneumonitis induced by anti-PD-1/PD-L1 therapy in non-small-cell lung cancer: occurrence and mechanism. Front Immunol.

[35] Jain V, Berman AT (2018). Radiation pneumonitis: old problem, new tricks. Cancers.

[36] Zhang Z, Zhou J, Verma V, Liu X, Wu M, Yu J, Chen D (2021). Crossed pathways for radiation-induced and immunotherapy-related lung injury. Front Immunol.

[37] Palma DA, Senan S, Tsujino K, Barriger RB, Rengan R, Moreno M, Bradley JD, Kim TH, Ramella S, Marks LB, De Petris L, Stitt L, Rodrigues G (2013). Predicting radiation pneumonitis after chemoradiation therapy for lung cancer: an international individual patient data meta-analysis. Int J Radiat Oncol Biol Phys.

[38] Tsujino K, Hashimoto T, Shimada T, Yoden E, Fujii O, Ota Y, Satouchi M, Negoro S, Adachi S, Soejima T (2014). Combined analysis of V20, VS5, pulmonary fibrosis score on baseline computed tomography, and patient age improves prediction of severe radiation pneumonitis after concurrent chemoradiotherapy for locally advanced non-small-cell lung cancer. J Thorac Oncol Off Publ Int Assoc Stud Lung Can.

[39] Khunger M, Rakshit S, Pasupuleti V, Hernandez AV, Mazzone P, Stevenson J, Pennell NA, Velcheti V (2017). Incidence of pneumonitis with use of programmed death 1 and programmed death-ligand 1 inhibitors in non-small cell lung cancer: a systematic review and meta-analysis of trials. Chest.

[40] Castanon E (2016). Anti-PD1-induced pneumonitis: capturing the hidden enemy. Clin Can Res Off J Am Assoc Can Res.

[41] De Velasco G, Je Y, Bossé D, Awad MM, Ott PA, Moreira RB, Schutz F, Bellmunt J, Sonpavde GP, Hodi FS, Choueiri TK (2017). Comprehensive meta-analysis of key immune-related adverse events from CTLA-4 and PD-1/PD-L1 inhibitors in cancer patients. Cancer Immunol Res.

[42] Chen Y, Liu X, Huang Z, Zhao K, Wang Y, Ren F, Yu J, Meng X (2021). Safety of thoracic radiotherapy after PD-(L)1 inhibitor treatment in patients with lung cancer. Cancer Med.

[43] Cousin F, Desir C, Ben Mustapha S, Mievis C, Coucke P, Hustinx R (2021). Incidence, risk factors, and CT characteristics of radiation recall pneumonitis induced by immune checkpoint inhibitor in lung cancer. Radiother Oncol J European Soc Therapeut Radiol Oncol.

